# Self-rated health impact of COVID 19 confinement on inmates in Southeastern of Europe: a qualitative study

**DOI:** 10.1186/s12889-023-17088-3

**Published:** 2023-11-07

**Authors:** Raquel Sánchez-Recio, Mario Samper-Pardo, Rebeca Llopis-Lambán, Bárbara Oliván-Blázquez, Marta Cerdan-Bernad, Rosa Magallón-Botaya

**Affiliations:** 1 Research Group on Health Services in Aragon (GRISSA), Department of Preventive Medicine and Public Health, Faculty of Social and Labor Sciences, University of Zaragoza, C/ Violante de Hungría (23), Zaragoza, 50009 Spain; 2 Institute for Health Research in Aragon (IIS Aragón), C. de San Juan Bosco, 13, Zaragoza, 50009 Spain; 3 Zaragoza Penitentiary Center, Autovía A-23, Km, 328, Zaragoza, Spain; 4 Department of medicine, Facultad de Medicina Edificio A, University of Zaragoza, Zaragoza, 5009 Spain; 5 Department of Psychology and Sociology, University of Zaragoza, Calle de Violante de Hungría, 23, Zaragoza, 2009 Spain

**Keywords:** Qualitative study, Inmates, COVID-19, Self-rated health

## Abstract

**Introduction:**

The COVID-19 pandemic necessitated the implementation of various measures within closed institutions like prisons to control the spread of the virus. Analyzing the impact of these measures on the health of inmates is crucial from a public health perspective. This study aimed to explore inmates’ subjective perception of the COVID-19 lockdown, the implemented measures, their physical self-perception, and their views on the vaccination process.

**Method:**

Between April 2021 and January 2022, 27 semi-structured individual interviews and 1 focus group were conducted with inmates in a prison located in northwest Spain. The interviews were conducted in person and audio-recorded. Thematic content analysis was employed, utilizing methodological triangulation to enhance the coherence and rigor of the results.

**Results:**

The analysis revealed two main themes and nine subthemes. The first theme focused on inmates’ perception of the implementation of protective measures against COVID-19 within the prison and its impact on their well-being. The second theme explored the pandemic’s emotional impact on inmates. All participants reported negative consequences on their health resulting from the measures implemented by the institution to contain the pandemic. However, they acknowledged that measures like lockdowns and mass vaccination helped mitigate the spread of the virus within the prison, contrary to initial expectations.

**Conclusion:**

COVID-19 and related measures have directly affected the health of inmates. To improve their health and minimize the impact of pandemic-induced changes, community participation and empowerment of individuals are essential tools, particularly within closed institutions such as prisons.

## Introduction

In late 2019, the disease SARS-CoV-2 (COVID-19), swept across Wuhan (China) at a lightening pace and was starting to spread rapidly around the world [1]. Consequently, in view of the scenario generated, different measures were taken around the world to contain the infection and to reduce the pressure on healthcare systems [2].

COVID-19 pandemic has impacted people’s health as a consequence of the psychological impact of lockdown and as a result job loss and family members being sick or deceased, into others [3]. Research conducted in China [3], Europe [4] and Spain [5] have shown that the COVID-19 lockdown has increased the prevalence of stress, anxiety, depression, somatization and the consumption of toxic substances such as alcohol and tobacco, which has led to an increase in the use of psychotropic drugs [6]. In these circumstances, emotional coping has been shown to be a key element in the process of understanding epidemics, in order to avoid psychological and social consequences [7]. There are many difficult situations to manage and understand with regards to ordinary people, so it could be deduced that they would be even more complex for people deprived of liberty (inmates), where communication with the outside social environment their families and others is limited [8].

COVID-19 has been particularly belligerent In certain social groups such as health professionals, the homeless and institutionalized people in nursing homes [9–12]. In this respect, studies have shown that the risk factors associated with infection high rates and mortality from COVID-19 in institutionalized people were older, with poor immune systems, with comorbidities (arterial hypertension, heart failure, diabetes mellitus, COPD, chronic renal failure and cancer), together with living in a closed institution where transmissibility is easier in a context of high contagiousness and virus virulence [13]. As well as nursing homes, prisons are also a closed institution. The impact of the COVID-19 pandemic inside prisons could be similar to that inside nursing homes, with the exception being that the predominant age group of people in prison is under 65 years of age. Inmates are considered more at risk and vulnerable to serious disease, generally due to a higher prevalence of immunosuppressive diseases, chronic diseases, heart disease and overcrowding [14]. There are several studies that analyse the impact of COVID-19 on prisons, specifically on how the measures adopted to contain the spread of COVID-19 has affected the health of inmates [3, 15], as well as how these have been accepted [16, 17]. Fovet et al. conducted a study in French prisons which showed that despite the psychological containment measures adopted during the pandemic, an increase in anxiety symptoms and psychiatric illnesses was observed [17]. In situations such as these, it is useful to investigate the impact on health occurring in specific populations, such as inmates, with the aim of designing action strategies that can help to reduce or mitigate the psychological impact that can occur as a result of periods of lockdown, such as those recently experienced.

In Spain, with the aim of establishing effective control and management within penitentiary institutions, a series of guidelines and recommendations were drawn up in the “Early Response Plan in a COVID-19 Pandemic Control Scenario” by the Ministry of Health, Social Services and Equality and jointly by the General Secretariat of Penitentiary Institutions (Table 1) [18, 19]. This document contemplates different strategies that prisons should implement depending on the timing of the pandemic and the incidence of COVID-19 cases among inmates. So far, the management of the COVID-19 crisis in Spanish prisons has been effective due to the low rate of infections within prisons. At the time of this study, there were a total of 221,337 inmates in Spain [20]. In this sense, as of April 30 of 2022, there have been 12 deaths as a result of COVID-19 in penitentiary institutions, as well as 120 hospital admissions, 2309 mild cases and 5304 asymptomatic cases. Similar results have been observed with equally effective efforts in other European prisons such as France, where measures were taken to contain the spread of the pandemic within their prisons, such as restricting external visits by both family members and outsiders, reinforcing hygienic measures, reinforcing staff and creating COVID corridors [17].

**Table 1:** Measures Adopted in Correctional Institutions to Contain the Spread of the COVID 19 Pandemic during Strict Confinement (March 15 to June 21, 2020) [18, 19]

1.- Access to the Institution by persons outside the prisons	Suspension of entry of any external people (professional or family).
2.-Admissions of new inmates of outside prisons from extra-penitentiary centres such as courts, hospitals or security forces.	People without symptoms: Quarantine (14 days maximum) in COVID module. People with symptoms: point 5.
3.-Penitentiary workers	Use of self-protection material and Pandemic containment measures. In case of compatible symptoms COVID referral to the Public Health System. Reduction of the number of workers on the prison to a minimum. Maintain a safety distance of 1–2 m between workers and do not form groups of people.
4.- Measures to protect inmates health	Information, awareness-raising and health education on suspected COVID cases and action measures.
5.- Measures to be adopted in COVID-19 cases	COVID19 cases not admitted to hospital, isolate (14 days maximum) in an individual cell with good ventilation, own bathroom and closed door. Only go out exceptionally taking the appropriate measures. If necessary due to lack of space, it will be considered joint isolation in cohort. Positive or suspicious inmates will only be carried out when strictly necessary, always taking the appropriate measures. If the release of a positive case is decreed, it must be communicated urgently to Public Health (PH) and Judicial authority.
6.-Measures to COVID19 contact	Cases contact COVID19 isolate in quarantine cell. In case of suspicion: restrict their movements, they will stay in an individual cell or joint insolation in cohort, with its own bathroom. Follow-up of COVID19 symptoms will be carried out. Any possible case that becomes a positive case should be notified to PH authority. If a suspect case is released from liberty, the PH and Judicial authority must be notified.

At public health level, due to the lack of studies in this population regarding the consequences of containment and COVID-19 containment measures, it is necessary to further investigate how this population deprived of liberty (inmates) has faced this type of lockdown and social isolation more than what they already experience on a daily basis. This is in order to evaluate the response given and to be able to implement improvements and strategies from a public health point of view for future situations that may arise.

For these reasons and due to the lack of research on the subject, the main objective of this study is to delve into the subjective perception of inmates about the period of COVID-19 lockdown, the measures presented and how it has affected their physical and emotional health (self-perception), as well as their perception of the COVID-19 vaccination process by means of a qualitative study that allows us to delve into individual subjectivity.

## Methodology

### Location and design of the study

This study, with a qualitative approach and a narrative design, aimed to find out the subjective perspective of inmates who have experienced the period of COVID-19 lockdown in prison, in addition to the COVID-19 vaccination process. This study was conducted in a prison in northeast Spain between April 2021 and January 2022. The prison in which this study has been carried out is a reference macro-prison in the northern part of Spain. In it there are pre-trial detainees awaiting trial and people who have already been sentenced with a final judgment. The inmates are classified in 16 residential modules according to their adaptation to the center and their dangerousness, plus an infirmary module. Spanish prisons are not watertight, i.e., an inmate in prison in Spain rotates through the different national centers depending on the proximity to his or her family, adaptation to the center and the person’s interest in attending training and labor reinsertion workshops. The prison in question has more than 350 cells. At the time of this research study, the prison had 1221 inmates (69 being women). The average age of the inmates was 38.8 (SD:18,4).

### Selection of participants

The purposive sampling method was used to invite people to participate in the study. The inmates invited to participate in the study were identified by the Research Group (RG), with the objective of selecting people who could provide information relevant to the objectives of the study. In addition, the initially selected inmates helped to identify other key informants for the study. Prior to the start of the study, a meeting was held with those who agreed to participate to verbally explain the objective of the study and the procedure to be followed, clarifying any doubts that arose. The information was given in writing so that they could read it later at their leisure. Those who agreed to participate were given the informed consent form to fill out and sign. Only one person did not agree to participate in the study. The researchers maintained the sampling until information saturation was reached to eliminate biased views and their subjective inferences regarding information saturation [21]. Once the information was collected, they provided the data collected from the participants to other experts. Sampling continued until both approved the adequacy of the information. Inclusion criteria for the study included: being in prison 6 months before the start of the study, in order to analyse the changes caused; understanding and speaking Spanish; not having severe cognitive impairment and being in their compliance centre. As for the exclusion criteria, those who refused to participate or did not sign the informed consent form could not participate in the study.

27 inmates (7 female), between ages of 30 and 60, were selected to participate in the study We conducted 20 phenomenological approach interview and 1 focus group (7 inmates, male and female). Sample description is in Table 2.

**Table 2:** Description of the sociodemographic data of the people participating in the study

	Male	Female	Total
Sex (n, %) ^1^	20 (74.1%)	7 (25.9%)	27 (100%)
Age (M, SD)^2^	43.2 (SD 10.8)	38.7 (SD 4.9)	38.8 (SD 18.4)
Nationality^3^ (n, %) ^1^	Spanish (15, 55.5%)	6 (22.2%)	21 (77.7%)
	Foreign (5, 18.5%)	1 (3.7%)	6 (22.2%)
Educational Level^4^ (n, %) ^1^	Low (19, 96.3) %	Low (7, 100%)	26 (9, 3%)
	Medium (0.0%)	Medium (0.0%)	
	High (1, 3.7%)	High (0.0%)	1 (3, 7%)
Number of sentences served with imprisonment	Repeat offenders	Repeat offenders	Repeat offenders
Years currently in prison (M, SD)^2^	11.11 (SD 9.8)	7.14 (SD 5.9)	9.1 (SD 7.9)
Rotation through penitentiary centers (n, %) ^1^	3 (15%)	0 (0%)	3 (15%)

1.-n: number of persons, % as a percentage of the total number of inmates included in the study. 2.-M: mean, SD: standard deviation. 3.-Foreign nationality refers to the other nationalities of the persons participating in the study. 4. Educational level: The level of education was calculated from the International Standard Classification of Education (ISCED) (the International Standard Classification of Education (UNESCO, 2012) and recoded into three categories: low (lower and secondary education), medium (high school and intermediate vocational training) and high (higher degrees and or careers). 5.- Number of sentences served with imprisonment: in this case the person was considered to be a recidivist when he/she had served more than one prison sentence

### Procedure for collecting information (conducting the interview)

In order to obtain as much information as possible in this study, it was considered important to use a mixed qualitative methodology that included semi-structured individual interviews and focus groups. All in-depth interviews were audio-recorded and conducted by three research nurses who had provided care in the correctional facility. Having an average length of service in the institution of 15 years, they had training in qualitative methodology and had previously been trained on how to correctly conduct the interview. he average duration of the interview was 30 to 90 min.

Previously, a bibliographic search was conducted on research on the impact of the COVID-19 on inmates [8, 13, 15, 17, 22–27]. Based on the results of this search, the guides for the individual interviews and focus groups were elaborated. Table 3 below presents the detailed development of the interviews and focus groups.

**Table 3:** Interview questions

	Questions to guide individual interviews and focus group discussions
1	Can you tell me how you felt during the COVID-19 lockdown?
2	What has bothered you most during lockdown and why?
3	How would you say prison lockdown has affected your physical and mental health?
4	Did you feel you were safe inside the prison?
5	How do you usually communicate with your family, and during the lockdown, did your family say anything to you about it?
6	In the module, was the atmosphere altered by this situation and the panorama that was experienced?
7	What was your experience of the external situation?
8	By what means did you obtain information about the situation that was taking place, i.e., about COVID?
9	What is your opinion on the efficacy of the vaccine against COVID-19, as well as on the vaccination process implemented in our country?

### Data analysis

To ensure the reliability of the data, the RG will be formed by different researchers with different profiles and levels of familiarity with the context in the data analysis process. The transferability of the results will be reinforced, as already described, through an adequate selection of participants, as they will be selected for their ability to provide information related to the research question, discourses and sufficient number to obtain information saturation. Finally, it should be noted that a naturalistic generalization approach will be taken into account in which the context of the study is described so that the readers of the study can judge the transferability of the results to other contexts. The transcription of the data will be imported into a qualitative data management software [28]. The data will be analyzed according to the grounded theory approach with the constant comparison method. Data analysis will be conducted in third phases, following the qualitative content analysis methodology described by Graneheim and Lundman (2004) [21]. First, the three researchers of the study read all transcripts and identified emerging themes and possible subthemes that were agreed upon in team meetings. Second, the researchers reviewed the transcripts and compared the themes and subthemes to verify that they were in line with the questions under study. Next, the participants’ quotations were extracted, and new themes were also identified. Thirdly, after a meeting of the research team, discrepancies and new themes were discussed. Interpretations of the data were discussed with the interviewers and a representative group of participants to obtain their consent [29]. This methodological triangulation increased consistency and rigor by combining multiple techniques and maximizing the breadth and depth of interpretations.

### Ethical considerations

The present study was sent for approval to the Research Ethics Committee of Aragón (CEICA) (PI21-341) and then, approval was obtained from the General Secretariat of Penitentiary Institutions. Subsequently, the participants were informed of the study and invited to participate, explaining the Informed Consent form and signing it if they agreed to participate in the study, as well as the recording of the voice-over. Participants were assigned a study ID prior to the in-depth interviews to ensure their anonymity and confidentiality. The linked name and study ID were stored on an encrypted computer and accessed only by the study PI.

## Results

A total of 24 subcategories were obtained and subsequently grouped into 9 categories, which after being analysed by different researchers were merged and reconverted into 2 main themes: (1) Implementation of protective measures against the spread of COVID-19 within the prison and its impact on inmates; (2) Impact of COVID-19 pandemic on the emotional well-being of the prison population.

**Fig. 1:**
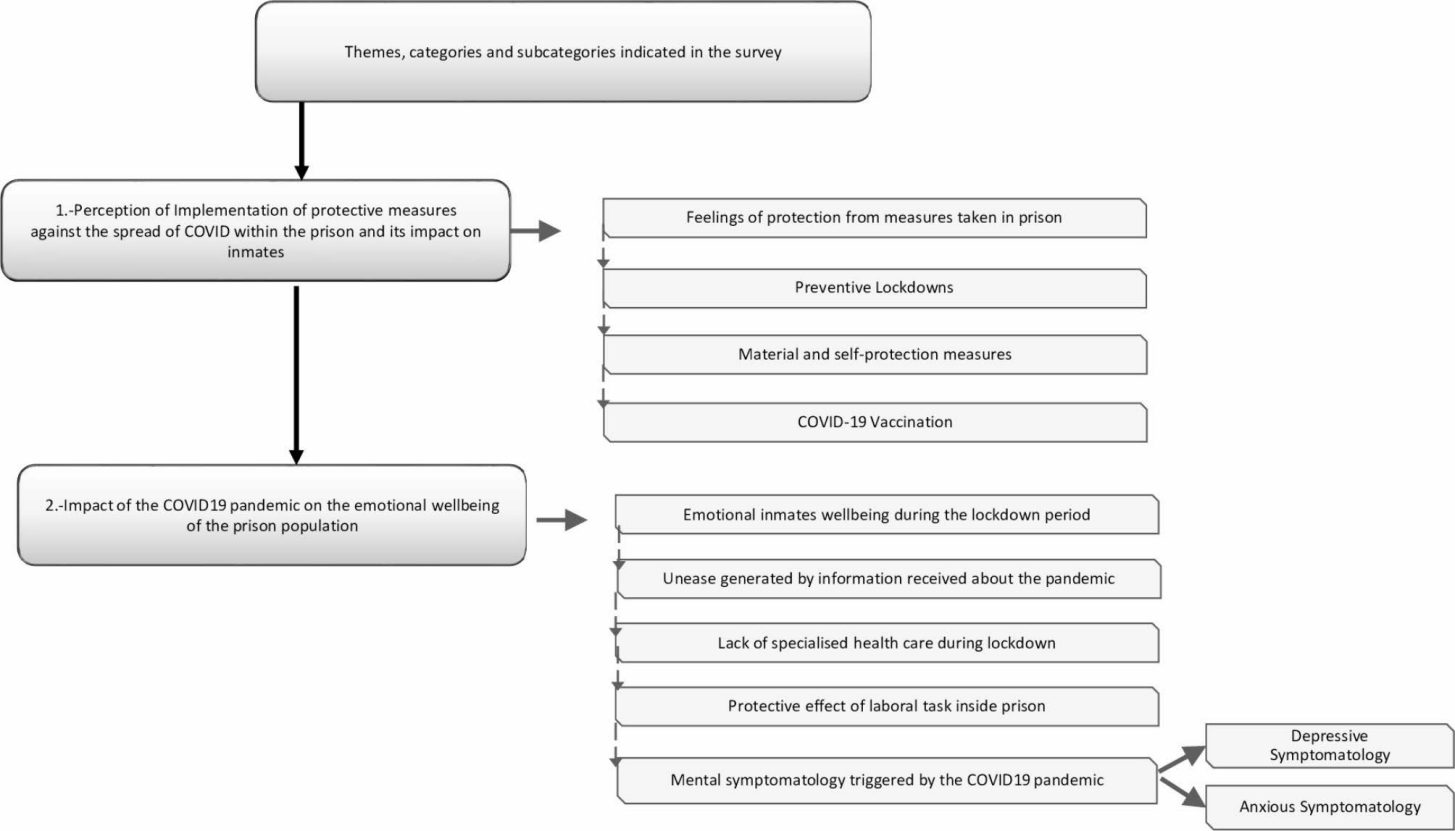
Themes, categories and subcategories of the information analysis

Figure 1 presents the themes, categories and subcategories indicated in this study.

### Perception of implementation of measures to protect against the spread of COVID-19 within a correctional facility and its impact on the prisoners

In the institution different protective measures were taken to contain the pandemic, such as the suspension of contact with outsiders, preventive lockdowns, use of self-protection measures, as well as the administration of COVID vaccines. In this regard and after analysing the information provided by the participants, we obtained four main sub-themes within this topic: 3.1.1 Feelings of protection in the face of measures adopted in prison; 3.1.2 Preventive lockdowns; 3.1.3 Self-protection material and measures; and 3.1.4 Vaccination against COVID-19.

#### Feelings of protection in the face of measures adopted in prison

The vast majority of the participants felt protected inside the prison by the measures taken to contain contagion. They felt they were in a bubble, recognizing that if the virus was not brought in by someone from outside, they would not catch it.

*“We were in a bubble… we have been safe because they cut communications, transfers and we did not communicate with anyone outside, we could not be safer.“*

(P6. Male, 30)

*“I got infected and was asymptomatic, if I had been on the street, I would have infected my father and my mother, and my mother is at risk, so it would have been worse. Here they have protected me and the rest of my colleagues.“*

(P20. Female, 37)

#### Preventive lockdowns

All inmates, despite understanding the positive effect of preventive lockdown, reported a negative effect on their health. For them, their preventive lockdown was similar to being in a solitary confinement situation (in this situation, the inmate lives in an individual cell and can only go out into the yard at certain times or alone or accompanied depending on the type of offence and living arrangements), i.e., isolated from their peers and only able to leave the cell to make one phone call a day to their families.

*“I was very upset, especially when I was locked down, it was the day I had a family visit and they suspended it. Seeing the family is to catch my breath to follow….“*

*(P8. Female, 42)*

*“Lockdowns meant being locked up, only going downstairs to make a phone call, or going down to the yard in shifts, to get food and come back up. You could stay more than 24 hours in the cell without moving or exercising, turning your head over. There were shifts for everything.”*

*(P11. Male, 51)*

*“The situation was worrying when we saw that more and more cases were reported in the block, first they closed the entire block, and we could not go out to the socio-cultural activities.“*

*(P25. Male, 63).*

In addition, a large majority of inmates reported a certain increase in conflict during periods of lockdown, highlighting the lack of mobility and the refusal of some inmates to be vaccinated, which generated a feeling of fear and insecurity.

*“We are all in the same block, we only have one women’s wing, that makes it difficult to live together, even more so during the pandemic where the fear, the hysteria, the rumours, the lack of contact with the family, made us more irritable, we argued more among ourselves… in the end this is like a schoolyard…”*.

*(P19. Female, 42)*

*“The atmosphere in general has been more tense… there are many factors that have influenced, we could not go outside… there was no movement between wings… you could feel the tension… it has been hard…”*

*(P26. Male, 36)*.

#### Material and self-protection measures

Personal Protective equipment (PPE) inside the prison was not given to inmates from the very beginning due to the lack of resources. A large number of the participants did speak of the uncertainty that this issue instilled in them and in particular of how to make use of them. It was also a challenge to raise awareness of the use of masks, as they themselves were reluctant to use it, especially in common areas where they were not in contact with working staff.

*“…actually, at the beginning, not PPE didn’t matter so much to us, since we didn’t mix with anyone from outside we were protected, the officials did wear masks so we were safe…”*.

*(P7. Male, 46)*

*“I work in nursing, I was the first person to intervene with a COVID case, they gave me everything, they put the EPIS on me, they explained to me how to take it off…. How scary it was.“*

*(P12. Male, 54)*

*“Here it is very rare that people, (we) wear masks, it is obligatory to wear them, we are given them with the hygienic material and if we need any more, we have the right to them, but almost nobody wears them, … we are not used to wearing them and even less in the yard where we are the only ones.“*

*(P21. Male, 35)*

#### COVID-19 vaccination

Regarding protective vaccines against COVID-19, all participants agreed to be vaccinated. All participants in this study were vaccinated at the time of the interviews. Of the few inmates who had not been vaccinated, none agreed to participate, given their refusal to anything related to the pandemic issue.

*“With vaccines I’m in favour of vaccinating, also earlier than on the street and I think that if in the end I catch it I’m going to pass it more mildly.“*

*(P1. Female, 37)*

*“Vaccines are necessary, anything that is to save lives is good, plus we all have to think about our health and the health of others, we protect ourselves, but we protect others.“*

*(P21. Male, 35)*

*“Even though I doubt it, I think it’s a good thing. I am very grateful that I was vaccinated inside the prison before people of my age were vaccinated outside. Getting vaccinated makes you go on furlough safer…I am very grateful.“*

*(P23. Male, 60)*

### Impact of the COVID-19 pandemic on the emotional wellbeing of the prison population

Life itself within the institution and the rules of operation have an important impact on emotional well-being. This fact has been accentuated through a pandemic where the lack of information resources, personal resources for coping and social contact such as the protection provided by the family was an important risk factor for emotional well-being. All the inmates interviewed reported having suffered some alteration in their emotional well-being during the pandemic, highlighting mainly the presence of depressive and anxiety symptoms. In this regard and after the analysis of the information provided by the participants, we obtained five main sub-themes within this topic: 3.2.1 Emotional well-being of the inmates during the lockdown period; 3.2.2 Lack of knowledge of COVID-19 by the inmates, 3.2.3 Lack of specialised care during lockdown, 3.3.4 Protective effect of work inside prison and 3.2.5 Mental symptomatology triggered by the pandemic due to COVID-19.

#### Emotional inmates’ wellbeing during the lockdown period

Almost all study participants reported a negative impact on their emotional well-being, due to the scenario generated inside the prison in relation to the COVID-19 pandemic.

*“The inactivity was hard, we could hardly go out, we had to go down to the courtyard by zones, you were afraid to meet people, everything hurt from doing nothing, my head was spinning, I admit I’m much worse, it’s been very hard.“*

*(P7. Female, 46)*

*“I recognize that this has had a great impact on me. What overwhelmed me was being confined as a preventive measure, it’s like living in solitary confinement and it’s bad, very bad, you don’t exercise, you don’t walk, you lie down all day, you eat what you shouldn’t, you change the rhythm of your sleep… in the end you resort to medication…”*.

*(P1. Female, 37)*

Likewise, when analysing the interviews of men and women, a greater impact on the emotional health of women was observed, with all of the participants reporting feeling worse. They were more concerned about the lack of communication and contact with their families, especially with their children. The lack of access to the outside complicated their daily lives.

*“It was hard, I watched TV, people died… I started to somatise, to have headaches… until today I had not felt like a prisoner, but when we were confined, I really felt like a prisoner. Not being able to touch your children is very hard, I needed support with treatment that I still continue to this day”.*

*(P2. Female, 32)*

In addition, a large percentage of the participants reported a sense of fear related to previous epidemics in prison, such as the onset of HIV and the fear of infectious diseases such as tuberculosis.

“I asked myself if this is like contagious tuberculosis, here we are very afraid of the bug, of the tupi, you know, people have died from the tupi, and this is like tuberculosis, isn’t it? Here in prisons we have compared Covid with the HIV epidemic when it came in, at that time we saw many people die….

(P4. Male, 48)

#### Unease generated by information received about the pandemic

All study participants reflected the impact on their emotional well-being of lack of information about COVID 19 and fear of the unknown, as well as the presence of anxious symptoms.

*“We didn’t know anything, everything was unknown and we were afraid, the little we talked to each other was scary.“*

*(P 9. Male, 34)*

*“We didn’t have enough information… there were many rumors, …. the family told us some things, others said other things, …. It made us nervous; we suspected bad things, we had internal conflicts ……”*.

*(P 5. Male, 45)*

The women were the ones who reported the most consequences in this regard, mainly reflecting that the lack of information mixed with the rumors generated in the courtyard and the news coming from outside (mainly from the families) generated a lot of anxiety, insomnia and internal conflict.

*“I was very afraid, …. My mother was telling me things from outside, in the courtyard they were talking, I was crying a lot, I didn’t know what was going on, I didn’t understand….“*

*(P 24. Female, 33)*

Excessive infodemic information - in some cases correct, in others not - makes it difficult for people to find reliable sources and trustworthy guidance when they need it. In the institution, the fact of being deprived of liberty increases the distrust related to the lack of information at that time about the virus, about what was going to happen and the fear of contagion. Many of the inmates who participated in the study reflected how the news they received from the outside related to COVID and catastrophic news increased their anguish and anxiety even more.

*“Why? … because we were there all day locked up, watching TV and of course the only thing we saw all day was death, more death, sick people, … all day on TV with the same thing, … and when they open the door to give you something you think about it and you get scared, I don’t know if it’s rational or not, but of course, you’re in there and you almost prefer that they don’t open the door…”*.

*(P3. Male, 31)*

*“I was very scared, my family told me what was on social networks, on TV, and I believed it all, there were many negative thoughts that you did not know how to stop, and the family told you something about what was said on the Internet and you were even more overwhelmed….“.*

*(P 6. Male, 30)*

#### Lack of specialized health care during lockdown

At the COVID-19 beginning, one of the first measures taken was to close the institution to anyone outside the prison, both family members and outside professionals, including health specialists such as psychiatrists. Almost half of the study participants reflected feelings of anxiety and nervousness as a consequence of the lack of contact with the specialist physicians, especially with psychiatry. One person was HIV+, 6 were being followed by psychiatry and one patient was being followed by nephrology for chronic renal insufficiency under pharmacological treatment. This led to a certain loss of adherence to treatment. Finally, when health conditions improved and extrapenitentiary consultations were resumed, patients reported an improvement when they resumed consultations with their specialists.

*“You take good care of us, but of course, being able to talk to the psychiatrist, being able to follow the indications that the specialists give us improves our situation, it is fundamental to be able to follow a correct treatment and contact with the specialists”.*

*(P15. Male, 54)*

*“I take my medication every month… not that I am convinced that I need it… the psychiatrist says it is necessary, but of course if I don’t talk to him… you explain it to me but I have my doubts… talking to him helps me”*.

*(P17. Male, 41)*

#### Protective effect of laboral task inside prison

Similarly, several participants reflected the protective effect of prison work on self-perceived health, as they acknowledged that keeping busy and helping fellow inmates had a positive effect on their health.

*“The work has been rewarding, despite everything I’ve been through. The anxiety and fear related to it has made me feel good working in the lockdown module. Helping the medical staff and my colleagues allowed me to spend more time in the yard playing sports and that made me sleep better.“*

*(P 2. Male, 32)*

*“I worked in nursing, I was one of the first to deal with COVID patients, even though I was afraid of catching the disease, helping health personnel and taking care of patients kept me busy, useful and tired, which allowed me to sleep better and feel better, I really felt that my work was useful for something and that encouraged me.”*

*(P3. Male, 41)*

#### Mental symptomatology triggered by the COVID-19 pandemic

The prevalence of psychiatric pathology in the institution is very high, as is the consumption of psychotropic drugs. 100% of the people participating in the study consumed psychotropic drugs and 6 of the participants were diagnosed with a serious mental disorder. The COVID 19 caused mental symptomatology such as depressive or anxious symptoms to increase in all study participants.

##### Depressive symptomatology

The participants, due to their situation of being deprived of their liberty, are more vulnerable to developing depressive symptoms. The situation resulting from the pandemic and the measures taken to contain its transmission meant that the inmates were even more isolated from the outside world, from their own families. This, together with the collective fear, the lack of information and the inability to understand everything that reached them, caused the vast majority of patients to experience an increase in depressive symptoms.

*“Everyone thought that many people were going to die, I was afraid, in fact I’m still afraid today, I haven’t been infected and I know that this has changed a lot but I’m still very afraid of getting infected…”*.

*(P4. Male, 48)*

*“It is an accumulation of thoughts, the head does not stop, you are here, my mother is at risk, I have younger siblings that I did not take care of them and now here … my mother cannot take care of them…. And me here… I’m still sinking more….I was taking care of my siblings because my mother couldn’t…”*.

*(P16. Male, 27)*

*“I have become depressed, because this afternoon I have been crying, without seeing my son, my family, my loved ones, I have lost my brother to covid too, and all this is weighing on me and I have been through so much and I have been sick thinking about my brother all day long…”*.

*(P5. Male, 47)*

Despite discomfort and fear generated, the possible consequences of the pandemic and the measures taken by the institution to contain contagion, the general inmates’ feeling are gratitude both to center and to staff in general and specifically to health professional. All people interviewed expressed their gratitude to both the institution and staff for their care during the pandemic and for preventing the virus from entering in a virulent form. They reported feeling as if they were in a bubble.

*“I am very grateful to health professional, you work very hard, you are attentive to all of us, you are not just one of us… even though sometimes we get angry you behave so well… you put up with us and give us the information you can… I talk to my colleagues and we all feel the same way”*.

*(P24. Female, 33)*

*“It sounds selfish, but we are safer than in the street, they did everything they could health professional, management, Madrid, the video calls helped to carry the feeling of loneliness for not seeing the family, gave an encouragement to be able to continue”*.

*(P4. Male, 48)*

*“In the lockdown module, the staff of the institution behaved very well with us, the officials, the social workers, the nurses who came every day to see us, it was only for a few minutes… but we were all waiting for that minute to see what they would tell us from the outside, to end the confinement… it was like living a first degree,… or worse….“*

*(P14. Male, 55)*

*“The video calls allowed me to see my children, to see that they were, well…I couldn’t hug them, I couldn’t touch them, but at least I could see them and see that nothing had happened…for a mother that’s fundamental”*

*(P13. Male, 43)*.

##### Anxious symptomatology

Likewise, anxious symptomatology was a constant in the reports of all the study, related to various facts such as not knowing what is happening, not being able to communicate with their families and the fact of being imprisoned.

*“We have been overwhelmed and scared because we were locked in there all day watching television and all we saw all day was death and more death. When they opened the door to give you something, you get scared.“*

*(P 17. Male, 41)*

*“Age has a lot of influence. The younger people were much more nervous, the older ones we used to make them more nervous… we needed to be calm… we had to help each other”.*

*(P20. Female, 37)*

“I was very anxious, I did not see my children, I could not touch them, it is horrible for a mother to be locked up here with a pandemic and not know what is happening with your children, I was very anxious, I called on the phone but of course I did not know if what they were saying was true…. When they put in the video calls the anxiety improved somewhat…but a mother needs to touch her children…. She needs to feel that they are well”.

*(P24. Female, 33)*

*“I was very anxious. It’s bad, one thinks the worst. I thought about being locked up all day within 4 walls with someone infected… you want to die… it’s bad”.*

*(P22. Male, 38)*

## Discussion

The management of the COVID-19 pandemic in closed institutions such as prisons has been a challenge for managers, health and non-health professionals, as well as for the inmates themselves. Numerous measures have been taken to contain the spread within prisons. Despite their great effectiveness, they have impacted the inmates’ health, who are already isolated from the outside world and with little contact with their families. In other hand, managing the use of PPE has also been a challenge for both staff and inmates, related to a significant lack of preventive health awareness among inmates. In this qualitative research with Spanish inmates, we have identified the perception of health impact during the COVID-19 pandemic. This study provides a basic understanding of the opinions and perceptions of inmates in this situation, helping to effectively manage future situations or new pandemic scenarios.

During the pandemic, to contain the spread of COVID within the prison, different lockdown measures were implemented. As a consequence of these measures, the participants in this study reported situations of stress and anxiety resulting from the lack of physical contact with their families and other inmates. They also described the lack of exercise and other activities as distressing, feeling that pre-trial lockdown was like living in solitary confinement or in a prison within the prison. Despite this, the inmates in our study reported a feeling of protection inside the institution, as if they were inside a bubble. In this sense, Pyrooz et al. (2020) [30] in their study conducted in a prison in Oregon, showed how inmates felt safe inside the prison for several reasons. On the one hand, they recognized that they could do little or nothing to prevent the entry of the pandemic inside the prison and on the other hand, the institution was actually taking measures to prevent its spread [30].

The vast majority of study participants understood the need for preventive lockdowns to contain the spread of the pandemic. However, despite this, all of the participants used the term that being in prison within the prison was like being in solitary confinement. This term has been previously described by Suhomlinova et al. [31]. This pandemic posed a real management challenge, with Spain having its management recognised as exemplary by the WHO [32]. However, it is a challenge to clearly define and implement an ethical and humane application of medical isolation and quarantine practices in prisons in order to curb transmission while also protecting the dignity of inmates [33]. Numerous institutions have been concerned about the welfare of inmates in relation to measures taken to contain the pandemic, such as preventive lockdowns. Despite the limitation of rights these measures entail, organizations such as the European Parliament [34] and the United Nations Office of Human Rights [35] have endorsed their effectiveness as a superior and exceptional measure to contain transmission. However, organizations such as the WHO [36] and CDC [37] pointed out the need to limit lockdowns to when it is strictly necessary and for the minimal amount of time, while increasing other types of communication such as telephone calls or even video calls, which were established in Spain. Along the same lines, a recent systematic review conducted with the objective of synthesizing what was known about the impact of COVID-19 on the health of incarcerated people concluded that there are necessary future research including the experiences of incarcerated people and correctional staff and effects of prolonged quarantine into others [38].

Another important point in the management of the pandemic was the delivery and correct use of PPE. The delay in delivering PPE has been reported in other studies on the impact of the pandemic in European [34] and American [33] prisons. This fact has risked causing an increase in the number of cases due to the risk of infection [39]. It is a major challenge to raise awareness of the correct use of PPE, such as the use of masks, due to the lack of awareness of their importance and their correct use, mainly in patients who are particularly difficult to adhere to therapy [40].

The participants in our study acknowledged the importance of the vaccine administration and thanked the institution for having vaccinated them before their age group outside the prison. In Spain, the recommendations established by the COVID-19 Vaccination Strategy of the Ministry of Health, Social Services and Equality [41] were followed for the administration of the COVID-19 vaccine. Despite the poor therapeutic compliance of prison inmates, vaccination rates in Spain are close to 95% [42], similar to non-prison population [43], complying with the WHO recommendations [40]. Strodel et al. (2021) [41] analysed the prioritization of the COVID-19 vaccine in prison inmates, reaching the conclusion that this prioritization is necessary from the public health point of view. These authors insist on the importance of continuing to prioritize and raise awareness in the administration of complete vaccines and booster vaccines in the groups included in the different strategies. Geana et al. (2021) [44] insist, together with the prioritization of vaccination in inmates, the prioritization of women, due to the significant health, social and labour vulnerability they face once they are released from prison.

Throughout this study, we have observed that several factors have influenced the emotional well-being of inmates during COVID-19. During the pandemic, it has been essential to combat fake news due to its significant impact on the population [45]. Similar results have been observed by other researchers in both inmates [8] and the non-prison population [46]. Schneeweis et al. (2023) has also investigated how press reports related to the handling of the pandemic in U.S. prisons have had a negative impact on the inmate population. These news reports have focused more on the measures taken to contain the pandemic than on how these measures affected their vulnerability, health status and human rights. To mitigate this impact and to favour the transmission of truthful information that helps to contain the emotional impact, it is important to create reliable information channels bidirectional with the prison and society at large. Inside the institutions, certain inmates can be trained as leaders in order to disseminate accurate information and be able to mitigate the negative effects of the same [47, 48].

One of the measures taken at the institution to contain the spread of the pandemic was to cut off all communication with the outside world, even temporarily suspending the presence of health specialists inside the prison. This measure has been similar to that taken in other European countries, demonstrating its effectiveness [27]. This has also highlighted the need for new forms of care, such as telemedicine, as Harrington et al. shown in their study in he Passachusetts jail during the COVID-19 pandemic [49]. These new care strategies can facilitate patient contact with specialists, especially in chronic cases such as psychiatric patients in whom both awareness of the disease and adherence to treatment are low [29]. This is despite the constant and dedicated work carried out by the nursing staff [42].

In our study, prison inmates reported an increase in symptoms of depression and anxiety related to the different measures taken to contain infection. These results are in line with those found in previous studies, demonstrating the presence of psychological disorders due to the COVID-19 pandemic, such as symptoms of anxiety and depression, in vulnerable groups such as prison inmates [17, 50]. Chimicz et al., in the same with our results, in Polish prison found that during the COVID1-9 pandemic a depressed mood predominant among the inmates, and in this period the mood was changing from more positive to more negative as consequence of the measures considered to contain the pandemic [50]. This fact was even more accentuated in the case of women, who mainly reported the lack of physical contact with their families as “the moment that helps you to carry energy to continue inside”. This fact had already been reflected previously in Spanish Penitentiary Institutions [18, 42]. Similar results have been found by Maycok and Dickson (2021) [51] in others European prisons, although they said that these results were not conclusive because other authors had not found the same results. Previous reports show there is an inequality regarding female inmates in prison in Spain [52]. A recent international scope review done in worldwide prisons concluded that in this sense, victims of domestic violence, refugees, ethnic minorities, and people from sexual and gender minoritie the group most vulnerable [53]. It is therefore necessary to continue to carry out studies to show whether the measures adopted and the mandatory reports have succeeded in reducing these gender inequalities in prisons. Recently, Garrihy et al. done a study in Irish Prison Service who concluded that inmates reported a drastic impact on their mental and physical health, and an impact about Prison health system relation with de insolation in the penological context [54].

Likewise, inmates said they were grateful to the staff in general and to the health personnel in particular. The health management of the pandemic in the world’s prisons and specifically in Spanish prisons has been a challenge. As with the rest of penitentiary health services [55], health personnel in institutions are short staffed and exhaustion is notable [33, 34]. Calls are being made from various quarters for more health personnel in the health services, especially in those centres working with vulnerable groups [36, 37]. This is not only to combat the current pandemic, but also to reduce social inequalities in health among vulnerable groups [56].

### Limitations and strengths

This study has some limitations. First, due to the small sample size of this qualitative research. In this regard however, it should be noted that the number of participants in this study was conducted to saturation discourse. Second, the participants in the study may not be representative of all inmates in Spain, although inmates may be transferred from one prison to another. Also, the COVID-19 measures to be taken in the prisons were different even within the same Autonomous Community. In addition to the national standards, each prison had the freedom to adopt its own measures according to its epidemiological situation. Thirdly, the investigators were educated and trained to conduct the study however in this particular study, there may be some bias related to data collection, data interpretation, and coding. Also, all interviewers were prison nurses, which could signify another interviewer bias with respect to the inmates and their regimental relationship with the institution. Fourth, we may have another bias with respect to the pandemic in that the age of the participants was between 30 and 60. We have not been able to explore the perception of those most at risk in the early hours. In the centre under study the average age of the inmates is 38.8 years and the percentage of people over 60 years was only 63 (1221 total). Despite all these limitations, we believe that this is one of the first Spanish studies to show how COVID-19 and the different measures implemented in institutions to contain its spread have had an impact on its self-perceived health, yielding interesting results for effective management of possible similar scenarios. Finally, studies like this one could also serve as a basis for future studies that will help us to understand the reality of the situation regarding the health of vulnerable groups such as the prison population.

## Conclusion

Knowing the perceptions about the impact on their self-perceived health of the COVID-19 pandemic and the different measures adopted to contain its spread in Spanish prisons can help institutional managers and health professionals to make effective decisions to improve the response to situations as complicated as the current pandemic. The inmates pointed out many factors that impacted their health, such as the lack of knowledge of the pandemic, the excess of information, the lack of contact with their families and the appearance of different anxious and depressive symptoms mainly related to the lockdowns (for them it was like being really prisoners and the prison inside the prison). They also recognized the difficulty and lack of perception in the preventive capacity of PPE, mainly the use of masks. Finally, they also pointed out as an important issue the gratitude to the staff of the institution, both health and non-health personnel. The management of the pandemic has been complicated by the lack of knowledge of the new. This management has been effective and exemplary in Spanish penitentiary institutions, and its effectiveness is internationally recognized.

## Data Availability

All data generated or analysed during this study are included in this published article.
